# Supplementary material for: From sequence to enzyme mechanism using multi-label machine learning
Source: BMC Bioinformatics. 2014 May 19;15:150. doi: 10.1186/1471-2105-15-150 (PMC4229970; doi:10.1186/1471-2105-15-150)
Supplement: Additional file 2 — Java code of ml2db. Additional file ml2db_code.tar.gz contains the Java source code to run the multi-label machine learning experiments and save the results to database. The code’s Javadoc is included. [file 1471-2105-15-150-S2.zip › additional file 2/ml2db/utils/doc/uk/ac/ed/inf/utils/database/package-frame.html]

uk.ac.ed.inf.utils.database


uk.ac.ed.inf.utils.database

Classes 
  
DbConn
  
DbConnectionSingleton
  
DbCreator
  
DbManaged
  
DbManager
  
DbManagerSingleton
  
DbReader
  
DbUtils
  
DbWriter
  
Managed
  
Manager
  
ResultSetUtils
  
SqlUtils
  
Table
  
TableColumn
  
TableCreator
  
TableManaged
  
TableManager
  
TableReader
  
TableRow
  
TableRowSaver
  
TableWriter
